# Analgesic Effectiveness of Magnesium Sulfate as an Adjuvant to Local Anesthesia in Symptomatic Irreversible Pulpitis: A Systematic Review and Meta-Analysis of Randomized Controlled Trials

**DOI:** 10.7759/cureus.94190

**Published:** 2025-10-09

**Authors:** Abdulwahab T Alenezi, Abdalla Alenezi, Salem Albadi, Abullah A Bakheet, Fawaz Alshammari, Abdulmuhsen F Alajmi, Mohammed Alkandari, Alya Almulla, Yousef Alqattan, Shahad Altaweel, Turki J Alshammari, Shouq Alshatti, Abdulaziz Almuthaybiri, Duaij Alsenafi

**Affiliations:** 1 Department of Dentistry, Saad Al-Abdullah Block 2 Polyclinic, Al-Jahra, KWT; 2 Department of Dentistry, Qasr Polyclinic, Al-Jahra, KWT; 3 Department of Dentistry, Kuwait National Guard Hospital, Kuwait City, KWT; 4 Department of Dentistry, Kuwait Institute for Medical Specializations, Kuwait City, KWT; 5 Department of Dentistry, Rumaithiya Polyclinic, Kuwait City, KWT; 6 Department of Dentistry, Omariya Polyclinic, Farwaniya, KWT; 7 Department of Dentistry, Sabah Al-Salem Polyclinic, Kuwait City, KWT; 8 Department of Dentistry, Jahra Polyclinic, Al-Jahra, KWT; 9 Department of Dentistry, Mubarak Al-Kabeer Eastern Medical Center, Mubarak Al-Kabeer, KWT; 10 Department of Dentistry, Al-Jahra Specialized Dental Center, Al-Jahra, KWT

**Keywords:** inferior alveolar nerve block (ianb), magnesium sulfate, meta-analysis, symptomatic irreversible pulpitis, systematic review

## Abstract

The inferior alveolar nerve block (IANB) is the most commonly used method for inducing pulpal anesthesia in patients with symptomatic irreversible pulpitis (SIP). Magnesium sulfate has been reported to increase the anesthetic effectiveness for endodontic procedures. Whether the adjuvant use of magnesium sulfate with IANB is effective in SIP patients remains uncertain. We performed an electronic search on PubMed, Scopus, Web of Science (WOS), and Cochrane CENTRAL from inception to September 2025 for randomized controlled trials (RCTs) assessing the adjuvant use of magnesium sulfate with IANB and local anesthesia compared to IANB with local anesthesia alone in patients with SIP. The primary outcome of interest was the pain scores following one and two hours post-procedure, while the secondary outcome was the anesthetic success rates. Standardized mean difference (SMD) and risk ratio (RR) with their 95% confidence intervals (CIs) were calculated using a random effects model. All analyses were performed using STATA 19MP. Seven RCTs comprising 398 patients were included in the final analysis. The adjuvant use of magnesium sulfate was associated with significant reductions in post-procedure pain scores at one hour (SMD = -1.97, 95% CI: -3.18 to -0.77, *p* < 0.001; I2 = 92.98%), without a significant difference at two hours post-procedure (SMD = -0.47, 95% CI: -1 to 0.06, *p* = 0.08; I2 = 0.00%). In addition, it was associated with higher rates of anesthetic success compared to the control group (RR = 1.61, 95% CI: 1.06 to 2.44, *p* = 0.02; I2 = 0.00%). The adjuvant use of magnesium sulfate showed a significant reduction in pain scores only at one hour following the procedure, without a significant difference at two hours. Moreover, magnesium sulfate had higher anesthetic success rates compared to the IANB alone. Further long-term RCTs with standardized protocols are recommended to confirm the current findings.

## Introduction and background

Effective management of pain is fundamental during endodontic practice, serving not only as a measure of patient comfort but also as a critical determinant of procedural success [1]. Enabling clinicians to perform root canal therapy without inducing pain, distress, and discomfort is crucial for patient-centered care. The standard dental local anesthesia technique has been the inferior alveolar nerve block (IANB), which is an indispensable technique for anesthesia during endodontic procedures. It helps mitigate the patient’s anxiety and fear, which often accompany endodontic procedures, thus fostering patient cooperation [2].

Achieving a sufficient degree of local anesthesia for mandibular posterior teeth is a significant challenge, particularly for patients with symptomatic irreversible pulpitis (SIP) [3]. However, clinical studies on patients with SIP have reported a failure rate of local anesthesia using the conventional IANB of 41-88% [4-6]. This failure might be attributed to several factors, including inflammation-induced tissue acidosis trapping the anesthetic agent, decreased concentration of local anesthetic solution (LAS) due to increased blood flow, the activating effect of inflammation on the peripheral free terminals of nociceptive neurons, the age and ethinicty of the patients, along with the associated central mechanisms, and prolonged inflammation triggers [7].

Some studies have also mentioned the process of central sensitization, a state of hyper-reactive response in the CNS driven by the upregulation of N-methyl-D-aspartate (NMDA) receptors, making the neurons more responsive to hyperalgesia and lowering the pain threshold, complicating efforts for adequate anesthesia [8]. Researchers have explored various strategies to tackle this clinical situation, including anesthetic adjuvants designed to minimize the impact of inflammation and the central sensitization process. As a natural antagonist of the NMDA receptor, magnesium sulfate (MgSO4) has emerged as a promising solution as an adjuvant to IANB for mandibular posterior teeth with SIP to increase the success rate of local anesthesia and endodontic procedures [9]. Neurophysiological studies have demonstrated that MgSO4 can block voltage-dependent ion channels, providing an antinociceptive effect [10].

Despite the increasing evidence regarding the use of MgSO4 as an adjuvant to IANB for local anesthesia in SIP patients, the lack of a collective, conclusive synthesis might hinder clinical decision-making. Therefore, we aimed to systematically collect and critically appraise the available published trials regarding MgSO4 as an adjuvant to IANB in patients with SIP.

## Review

Materials and methods

We followed the Preferred Reporting Items for Systematic Reviews and Meta-Analyses (PRISMA) [11] and the guidelines from the Cochrane Handbook of Systematic Reviews and Meta-analyses of Interventions [12] while conducting this systematic review and meta-analysis.

Literature Search and Screening

We performed a comprehensive electronic search on PubMed, Scopus, Cochrane CENTRAL, and Web of Science (WOS) from inception until September 2025 for relevant randomized controlled trials (RCTs) using the following search strategy: ("irreversible pulpitis" OR "symptomatic pulpitis") AND ("inferior alveolar nerve block" OR "IANB" OR "mandibular block") AND ("magnesium sulfate" OR "MgSO4"). The detailed search strategy adjusted for each database is shown in Table 1. In addition, we performed forward and backward citation analyses for the potentially included studies to ensure we included all relevant studies. The screening process was performed in a two-step fashion. First, we conducted title and abstract screening to identify eligible studies, and then full-text screening was done to assess whether the potential included studies met our eligibility criteria.

**Table 1:** Detailed search strategy for each database.

Database	Search terms	Search field	Search results
PubMed	("irreversible pulpitis" OR "symptomatic pulpitis") AND ("inferior alveolar nerve block" OR "IANB" OR "mandibular block") AND ("magnesium sulfate" OR "magnesium sulphate" OR MgSO4)	All fields, English	4
Cochrane	("irreversible pulpitis" OR "symptomatic pulpitis") AND ("inferior alveolar nerve block" OR "IANB" OR "mandibular block") AND ("magnesium sulfate" OR "magnesium sulphate" OR MgSO4)	All fields, English	11
WOS	("irreversible pulpitis" OR "symptomatic pulpitis") AND ("inferior alveolar nerve block" OR "IANB" OR "mandibular block") AND ("magnesium sulfate" OR "magnesium sulphate" OR MgSO4)	All fields, English	3
SCOPUS	("irreversible pulpitis" OR "symptomatic pulpitis") AND ("inferior alveolar nerve block" OR "IANB" OR "mandibular block") AND ("magnesium sulfate" OR "magnesium sulphate" OR MgSO4)	Title, abstract, keywords, English	5

Eligibility Criteria and Outcomes

We included all studies that met the following pre-defined eligibility criteria: 1) patients with symptomatic irreversible pulpitis; 2) the intervention was the adjuvant use of magnesium sulfate in addition to IANB with local anesthesia; 3) the control group received IANB with local anesthesia only; and 4) randomized controlled trials. Conversely, we excluded studies that did not use magnesium sulfate as an adjuvant therapy, conference abstracts, or unpublished data.

The primary outcome of interest was the assessment of pain scores following the intervention, evaluated by the Heft-Parker visual analogue scale (HP-VAS) and the numerical VAS scale. The HP-VAS assessment tool rated each patient using a 170-mm marked line divided into four categories with various terms describing the level of pain. The four categories were no pain (0 mm), mild pain (1 to 54 mm), moderate pain (55 to 113 mm), and severe pain (114 to 170 mm) [4]. In addition, the numeric VAS tool categorizes pain on a 10-point scale, where “0” indicates no pain and “10” indicates the worst pain ever [13]. The secondary outcome of interest was the anesthetic success rate.

Quality Assessment

We assessed the quality of the included studies using the risk of bias assessment tool for RCT version 2 proposed by the Cochrane risk-of-bias tool for randomized clinical trials (ROB-2) [14]. Version 2 comprises five domains assessing the bias arising from the randomization process, deviations from the intended interventions, missing outcome data, measurement of the reported outcomes, and selection of the reported results. Each domain was classified as low-risk, high-risk, or having some concerns, with an overall risk of bias assessment. Any discrepancies were resolved by discussion.

Data Extraction and Statistical Analysis

We used a standardized Excel sheet (Microsoft Corp., USA) to extract and present the necessary data from the included studies and patients as follows: 1) baseline characteristics of the patients from included studies, including sample size, age, gender, and baseline pain score; 2) summary characteristics of the included studies, including study design, country, patient characteristics, intervention, control, pain scale, IANB technique, and follow-up; 3) the ROB-2 five domains; and 4) the assessed outcomes.

Continuous outcomes were extracted as post-intervention mean values, together with standard deviations (SDs) and the total number of included patients. Dichotomous data were extracted as event/total and pooled as risk ratios (RRs) with 95% confidence intervals (CIs). All continuous data were pooled and analyzed as standardized mean differences (SMDs) with 95% CIs using the DerSimonian-Laird random-effects model. Heterogeneity was assessed using the Cochrane Q test, and the I² measure was determined across all studies. A p-value less than 0.05 and an I² value ≥50% were considered indicative of significant heterogeneity among the included studies. The packages “meta esize” and “meta forest plot” were used in STATA 19MP software (StataCorp LLC, Texas, USA) to pool the effect estimate and the corresponding 95% CI.

Results

Literature Search

**Figure 1:**
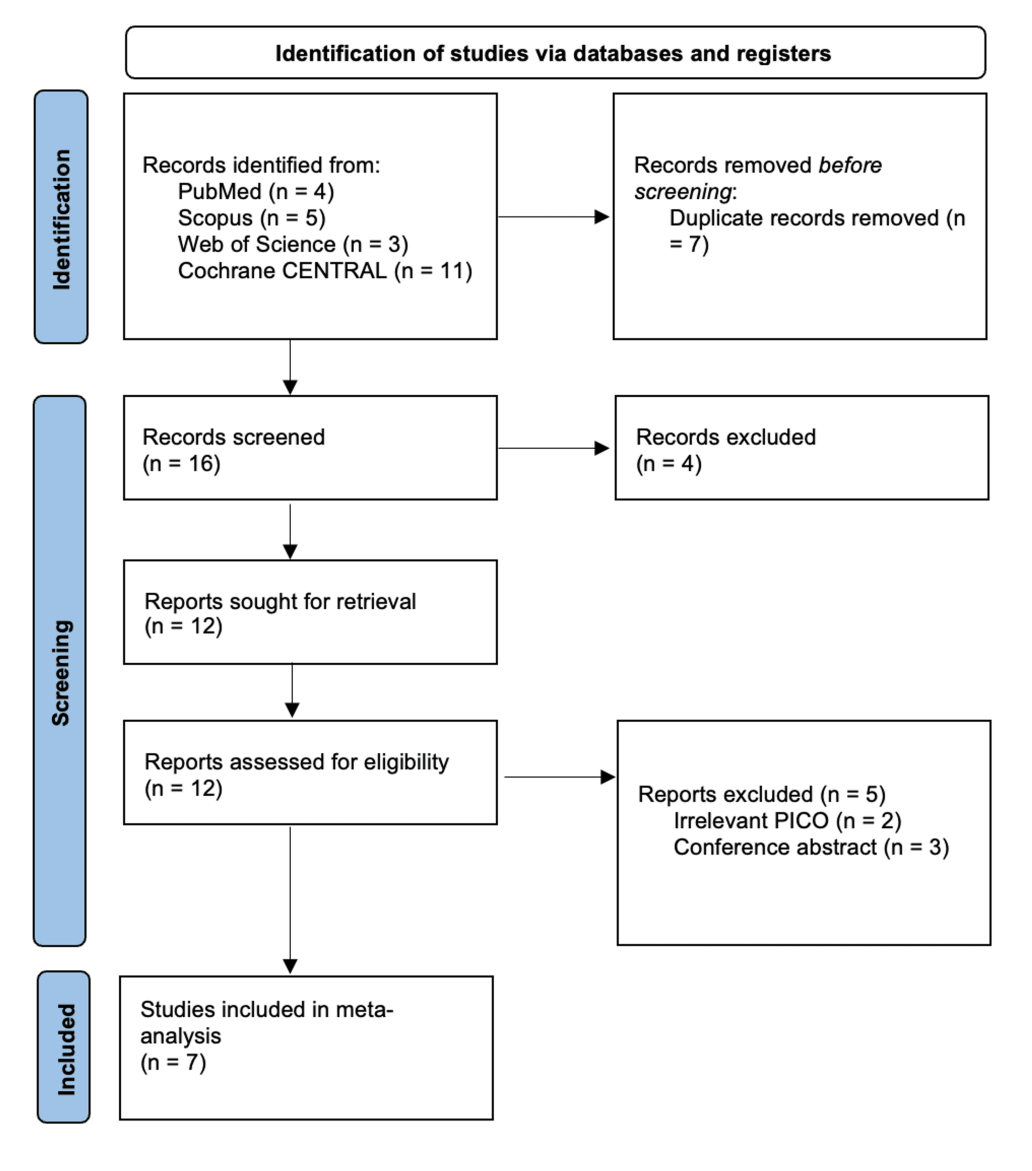
Preferred Reporting Items for Systematic Reviews and Meta-Analyses (PRISMA) flowchart of the study selection process.

Our comprehensive search of the databases yielded 23 articles, of which 11 were excluded after duplicate removal and title-abstract screening. Only 12 articles were eligible for full-text screening, and seven RCTs were finally included in the systematic review and meta-analysis [15-21]. The selection process is presented in the PRISMA flow diagram (Figure 1).

Baseline Characteristics and RoB Assessment

The seven RCTs included a total of 398 patients, with a mean age of 33.2 years. A total of 210 (52.7%) patients were allocated to magnesium sulfate, while 188 (47.3%) patients were allocated to the control group. Detailed baseline characteristics of the patients and a summary of the included studies are stated in Table 2 and Table 3.

**Table 2:** Summary characteristics of the included RCTs. RCT: randomized controlled trial; IANB: inferior alveolar nerve block; SIP: symptomatic irreversible pulpitis; HP-VAS: Heft–Parker visual analogue scale

Study ID	Country	Study design	Sample size	Patient characteristics	Intervention	Control	Pain scale	IANB technique	Follow-up schedule
Shetty, 2015 [19]	India	RCT	100	Adults aged 18–60 with SIP in a mandibular molar	IANB + 1 mL of 50% MgSO₄.	IANB	HP-VAS	Standard IANB with 1.8 mL of 2% lidocaine with 1:100,000 epinephrine.	During access cavity preparation and 15 mins post-IANB
Chandrasekaran, 2020 [16]	India	RCT	42	Patients aged 20–60 with SIP and symptomatic apical periodontitis in mandibular molars.	Group A: IANB + 75 mg MgSO₄. Group B: IANB + 150 mg MgSO₄.	IANB	HP-VAS	IANB was administered at a rate of 1.5 mL over 60 seconds.	2, 6, 12, 24, and 48 hours post-operatively
Mousavi, 2020 [18]	Iran	RCT	68	Adults aged 18–60 with SIP in a mandibular molar	IANB + 1% MgSO₄.	IANB	HP-VAS	Standard IANB was administered slowly over 100 seconds.	During access cavity preparation and 15 mins post-IANB
Mekhimar, 2023 [17]	Egypt	RCT	48	adults aged 18–55 with SIP in mandibular molars	IANB + 1% MgSO₄.	IANB	HP-VAS	Conventional IANB was administered slowly over 100 seconds.	During access cavity preparation and 15 mins post-IANB
Sitharthan, 2024 [20]	India	RCT	86	Patients aged 18–45 with SIP of a mandibular molar.	IANB + 0.2 mL of 10% MgSO₄.	IANB	10-point VAS	IANB was administered at a rate of 1.8 mL over 60 seconds.	During access cavity preparation and 15 mins post-IANB
Priyadharshini, 2018 [21]	India	RCT	12	Patients with moderate to severe pain diagnosed with SIP of mandibular posterior teeth	IANB with 1 ml of magnesium sulphate 50%	IANB	HP-VAS	IANB was administered by a single operator using a 2 ml syringe and 26-gauge 31 mm long needle.	During access cavity preparation
Amin, 2023 [15]	Egypt	RCT	42	Patients with symptoms of SIP related to lower molars	IANB with 0.18 ml of MgSO4 solution 10% weight-volume	IANB	HP-VAS	IANB was administered at a rate of 1.8 mL over 60 seconds.	During access cavity preparation

**Table 3:** Baseline characteristics of the included patients. NA: not available

Study ID	Arm	Sample size	Age, y		Gender, n (Female/Male)	Baseline pain score	
			Mean	SD		Mean	SD
Shetty, 2015 [19]	Intervention	50	33.48	3.8	31 / 19	135.6	10.2
	Control	50	31.8	4.4	27 / 23	136.96	9.5
Chandrasekaran, 2020 [16]	Intervention 1	14	NA	NA	8 / 6	124.57	24.422
	Intervention 2	14	NA	NA	6 / 8	122.57	14.064
	Control	14	NA	NA	10 / 4	122.64	18.17
Mousavi, 2020 [18]	Intervention	34	36.5	12.8	21 / 13	141.5	15.71
	Control	34	40.82	15.1	21 / 13	133.03	12.07
Mekhimar, 2023 [17]	Intervention	24	35.88	10.83	18 / 6	NA	NA
	Control	24	33.92	9.56	15 / 9	NA	NA
Sitharthan, 2024 [20]	Intervention	43	30.37	9.02	26 / 17	7.48	0.85
	Control	43	26.39	7.72	25 / 18	7.83	0.94
Priyadharshini, 2018 [21]	Intervention	6	NA	NA	NA	105.3	13.75
	Control	6	NA	NA	NA	98.5	10.57
Amin, 2023 [15]	Intervention	25	37	10	NA	140	14
	Control	17	40	12	NA	145	12

**Figure 2:**
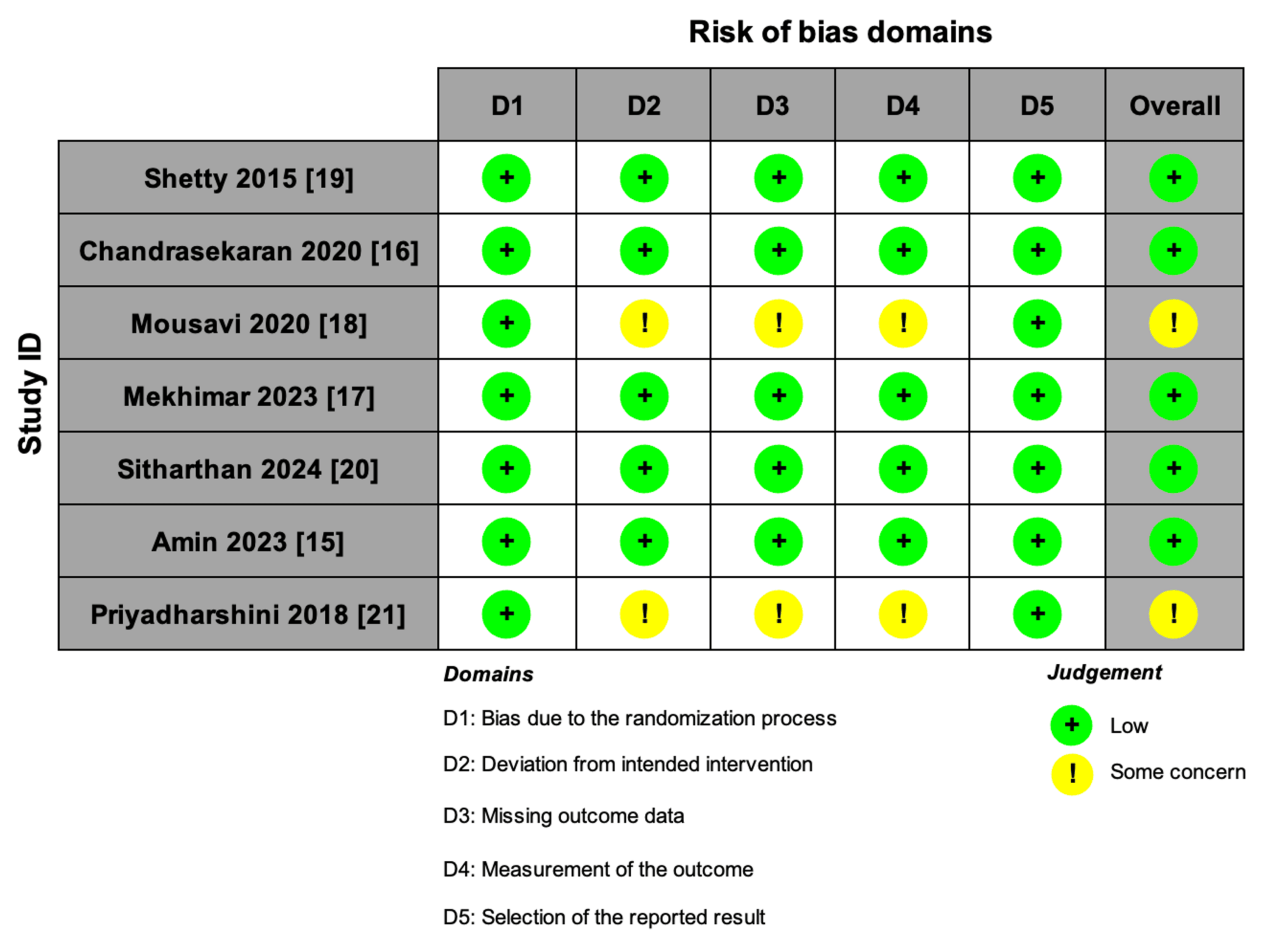
Risk-of-bias assessment of the included RCTs. Sources: [15-21] RCT: randomized controlled trial

All included RCTs were assessed using the ROB-2 tool, and all of the included studies had an overall low RoB, except for Mousavi et al. [18] and Priyadharshini et al. [21], which had some concerns mainly regarding deviations from intended interventions, missing outcome data, and measurement of the reported outcomes (Figure 2).

Outcomes

**Figure 3:**
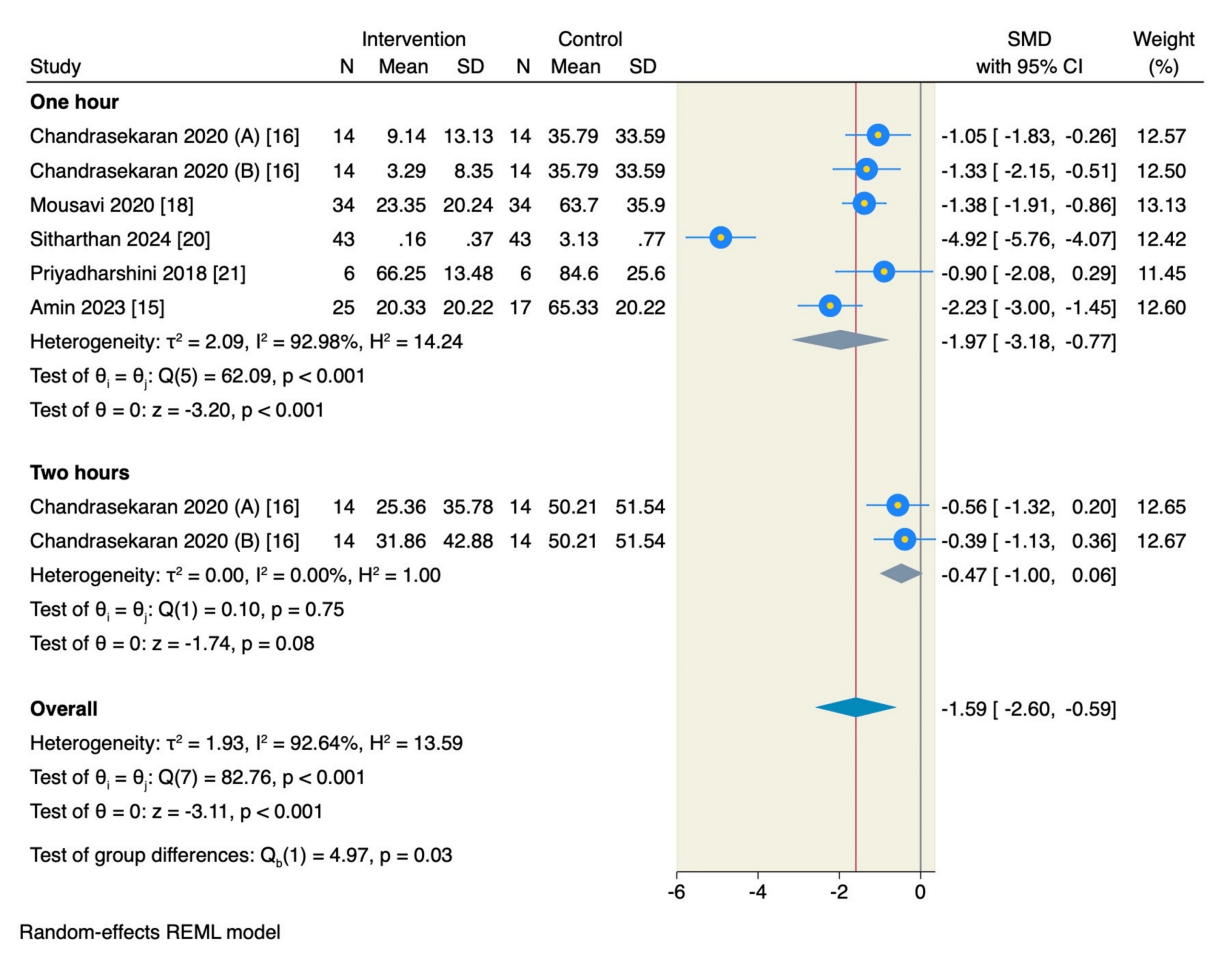
Random-effect model of the post-procedure pain score. Chandrasekaran (2020) (A) [16] represents the group receiving IANB + 75 mg MgSO₄, whereas Chandrasekaran (2020) (B) [16] represents the group receiving IANB + 150 mg MgSO₄.

Post-procedure pain score: Five RCTs assessed the pain score one hour following the procedure, of which the pooled estimate favored the intervention of magnesium sulfate to decrease post-procedure pain compared to the control group (SMD = -1.97, 95% CI: -3.18 to -0.77, p < 0.001; I² = 92.98%). However, only one study reported the pain score two hours post-procedure, and the pooled estimate showed no significant difference between the intervention and control groups (SMD = -0.47, 95% CI: -1 to 0.06, p = 0.08; I² = 0.00%), as shown in Figure 3.

**Figure 4:**
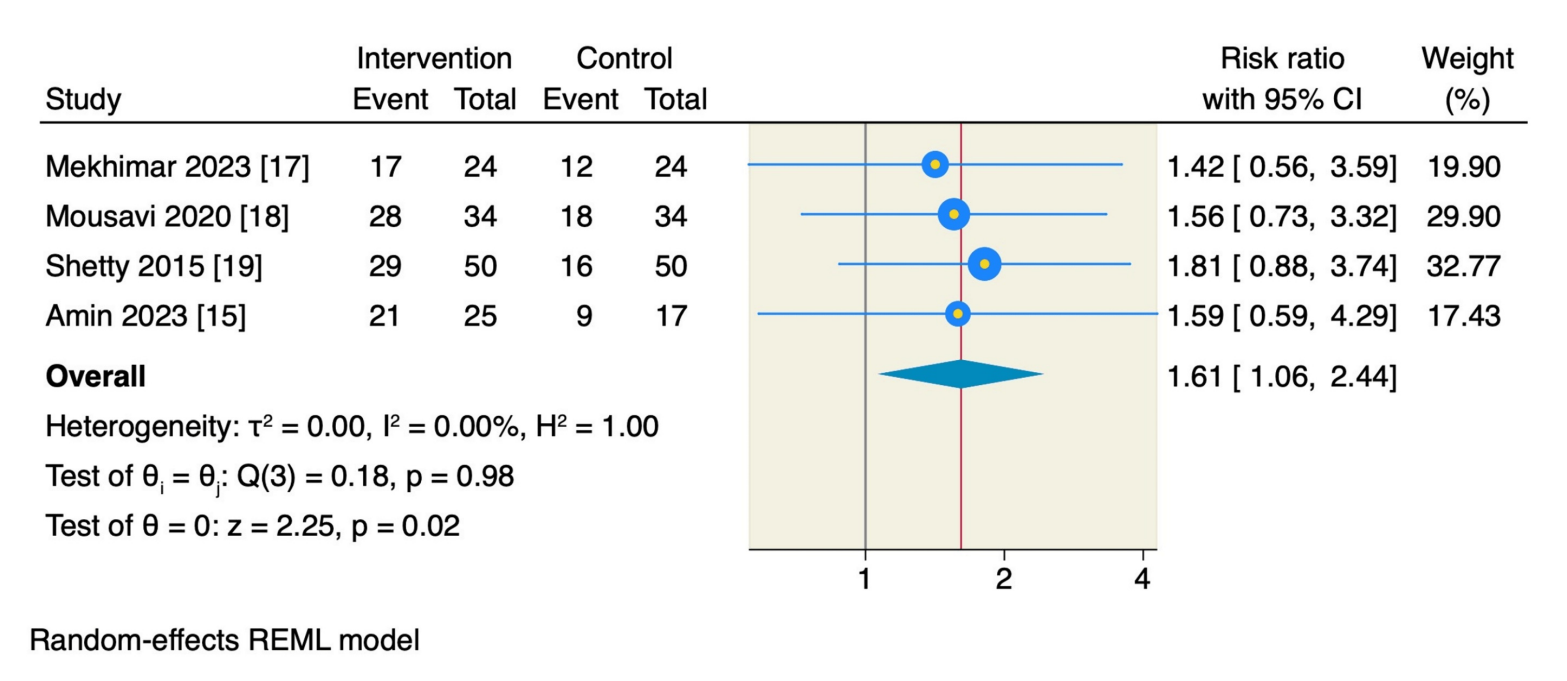
Random-effect model of anesthetic success rate.

Anesthetic success rate: Four RCTs reported the anesthetic success rate, with the rate significantly higher in the magnesium sulfate group: 45.2% (95 of 210 patients) compared to 29.25% (55 of 188 patients) in the control group. The pooled estimate showed a significant increase in the anesthetic success rate among patients in the magnesium sulfate group compared to the control group (RR = 1.61, 95% CI: 1.06 to 2.44, p = 0.02; I² = 0.00%), as shown in Figure 4.

Discussion

This systematic review and meta-analysis of 398 patients represents the first and most comprehensive study to compare the adjuvant use of magnesium sulfate in addition to IANB and local anesthesia in patients with systemic irreversible pulpitis. Our analysis showed that the addition of magnesium sulfate to IANB was significantly associated with lower pain scores following the procedure and higher anesthetic success rates compared to IANB with local anesthesia only.

Pain during surgical procedures is a complex phenomenon that requires multidimensional assessment. In patients with systemic irreversible pulpitis, the transmission of pain from the periphery to the central nervous system is mediated through voltage-gated sodium channels [22]. Substances such as bradykinin and prostaglandin E2 are the key modulators of the stimulation of the nociceptors at the neurons, and in cases of pulpal pain, the stimulation of these nociceptors exhibits a high degree of resistance to the administration of local anesthetics [23]. It is proposed that higher failure rates of local anesthetics during pulpal inflammation might be linked with an increase in the resistant sodium channels [24]. Magnesium sulfate is currently being used in daily clinical practice as an adjuvant general anesthetic solution and is being validated in various medicinal aspects [25], with previously documented solutions to increase anesthetic efficacy [26].

Our study found a significant reduction in pain scores and higher anesthetic success rates among patients allocated to the magnesium sulfate group compared to the control group. Magnesium sulfate is a natural physiological antagonist to voltage-gated channels and plays a key role in anti-nociception by blunting NMDA receptor activations and thus obstructing the excitatory neuronal fibers [27]. Moreover, it can be used as a sole analgesic agent; however, the recent use of magnesium as an adjuvant to established anesthesia has increased the synergy of the anesthetic solutions [10]. Therefore, it can be used as a dose-dependent solution to increase the onset, duration, anesthetic efficacy, and post-operative analgesia. Similar to our findings, Chandrasekaran et al. [16] and Mousavi et al. [18] reported higher anesthetic efficacy and lower post-operative pain following the adjuvant use of magnesium sulfate in patients with irreversible pulpitis. The anesthetic efficacy was assessed by measuring the levels of pain during the preparation of the access cavity using HP-VAS [28]. This method was based on the standard reporting of Aggarwal et al. [29] and Simpson et al. [30], which defined anesthetic efficacy as the tooth without pain (HP-VAS score equal to 0 mm) or with mild pain (HP-VAS rating ≤ 54 mm). On the other hand, the variability of the magnitude of effect estimates across the included studies might be attributed to the doses of magnesium sulfate used, which ranged from 75 mg to 170 mg; however, all studies that pre-specified doses of magnesium sulfate showed higher anesthetic efficacy and lower post-operative pain compared to the control group.

In addition, Shetty et al. [19] recruited patients with symptomatic irreversible pulpitis and allocated them to magnesium sulfate USP 50% one hour before the administration of conventional IANB solution, reporting higher IANB effectiveness in the magnesium sulfate group compared to the control group. However, the patients in the magnesium sulfate group were allocated to receive two injections, which could raise potential risks and higher rates of patient dissatisfaction [19]. Therefore, the use of magnesium sulfate as an adjuvant to local anesthesia in the same injection for IANB would be more beneficial.

The use of magnesium sulfate is inexpensive and safe in combination with other local anesthetic solutions such as lidocaine or mepivacaine [31]. The biological basis of magnesium sulfate for NMDA antagonism and Ca channel blockade effects can be utilized in cases of prolonged states of inflammation that might cause a significant increase in the number of NMDA receptors (hyperalgesia), which could result in a significant reduction of the intensity of pain stimulation [31]. Additionally, magnesium sulfate appears to be safe with no notable adverse events; however, it should be noted that higher doses of magnesium sulfate could result in potential risks and dissatisfaction [28].

Our study is the most comprehensive report; however, some limitations should be addressed before generalizing the findings of the current study. First, the different doses of magnesium sulfate could raise some potential differences regarding its proper use as an adjuvant therapy to IANB and local anesthesia. Despite no notable effect of the dose on our pooled estimates, a standardized approach should be generalized with more consistent findings over the follow-up duration. Second, the sample size of the included studies was relatively small, indicating the need for larger studies to further consolidate the benefits of magnesium sulfate as an adjuvant therapy. Additionally, the follow-up duration among the included studies was very short, with most studies reporting data only one hour post-intervention, and only one study reported up to 48 hours. Despite consistent findings up to 48 hours, more studies with longer follow-up durations are needed to consolidate the findings over a longer period. While the studies used standardized protocols, some variations in the clinical setting were noticed, especially regarding the initial pain severity of the included patients, which could be a source of heterogeneity.

## Conclusions

Our meta-analysis of seven RCTs and 398 patients reported higher anesthetic success rates and lower pain scores following the administration of magnesium sulfate as an adjuvant therapy with IANB and local anesthesia compared to the control group. The current findings open the door for orthodontists to consider the addition of magnesium sulfate to IANB in patients with symptomatic irreversible pulpitis. Further RCTs with standardized protocols are needed to validate these findings.
